# Exercise-based cardiac rehabilitation in stable angina pectoris: a narrative review on current evidence and underlying physiological mechanisms

**DOI:** 10.1007/s12471-023-01830-y

**Published:** 2023-11-20

**Authors:** Joyce M. Heutinck, Iris A. de Koning, Tom Vromen, Dick H. J. Thijssen, Hareld M. C. Kemps

**Affiliations:** 1 Department of Medical BioSciences, Radboud University Medical Center, Nijmegen, The Netherlands; 2 Department of Industrial Design, Eindhoven University of Technology, Eindhoven, The Netherlands; 3 Department of Cardiology, Maxima Medical Centre, Veldhoven, The Netherlands; 4 Research Institute for Sport and Exercise Sciences, Liverpool John Moores University, Liverpool, UK

**Keywords:** Angina stable, Exercise therapy, Cardiac rehabilitation, Chronic coronary syndrome, Endothelium, Vascular physiology

## Abstract

Stable angina pectoris (SAP) is a prevalent condition characterised by a high disease burden. Based on recent evidence, the need for revascularisation in addition to optimal medical treatment to reduce mortality and re-events is heavily debated. These observations may be explained by the fact that revascularisation is targeted at the local flow-limiting coronary artery lesion, while the aetiology of SAP relates to the systemic, inflammatory process of atherosclerosis, causing generalised vascular dysfunction throughout the entire vascular system. Moreover, cardiovascular events are not solely caused by obstructive plaques but are also associated with plaque burden and high-risk plaque features. Therefore, to reduce the risk of cardiovascular events and angina, and thereby improve quality of life, alternative therapeutic approaches to revascularisation should be considered, preferably targeting the cardiovascular system as a whole with a physiological approach. Exercise-based cardiac rehabilitation fits this description and is a promising strategy as a first-line treatment in addition to optimal medical treatment. In this review, we discuss the role of exercise-based cardiac rehabilitation in SAP in relation to the underlying physiological mechanisms, we summarise the existing evidence and highlight future directions.

## Introduction

Stable angina pectoris (SAP) is caused by myocardial ischaemia, with symptoms typically being provoked during increased cardiac demand, such as during physical activity. Several causes can lead to the supply-demand mismatch, with obstructive coronary artery disease being the most common. Although there may also be a role for microvascular dysfunction, we have primarily focussed on the macrovasculature in this review. Currently, initial treatment consists of preventive and antianginal medication to reduce symptoms and to improve quality of life and long-term morbidity and mortality. When symptoms persist, coronary angiography and subsequent revascularisation (i.e. percutaneous coronary intervention (PCI) or coronary artery bypass grafting) are commonly applied. Approximately 35% of all (~40,000) PCIs in the Netherlands are performed in patients with stable coronary artery disease [1]. Although revascularisation can result in rapid symptom relief [2], long-term prognostic advantages seem limited [2–5] and are increasingly debated. A recent randomised controlled trial (RCT) compared revascularisation with optimal medical therapy in 5179 SAP patients with myocardial ischaemia, and found no between-group differences in mortality or major adverse cardiovascular events (MACE) after a median 3.2-year follow-up [2]. Furthermore, a meta-analysis of 14 trials also showed no difference in all-cause mortality and cardiovascular mortality between revascularisation or optimal medical therapy alone after a median follow-up of 4.5 years [4]. Although this meta-analysis found more freedom of angina in favour of revascularisation, a more recent meta-analysis showed no difference between groups after 12-months of follow-up [5]. Finally, the ORBITA trial, the only double-blind study comparing PCI versus a sham procedure in SAP, showed no difference in symptoms and exercise capacity in 200 patients after a 6-week follow-up [3]. These observations question the value of revascularisation for relief of symptoms, even in the short term, and for prevention of cardiovascular events in the longer term in patients with SAP.

The lack of clinical benefit for revascularisation strategies may be explained by its focus on treating local flow-limiting coronary artery lesions, while the aetiology of SAP relates to the systemic, inflammatory process of atherosclerosis. This dynamic and gradual process involves chronic low-grade inflammation, not only causing the focal development of a flow-limiting stenosis, but also generalised vascular dysfunction and non-obstructive coronary lesions [6]. As a direct consequence, the risk of MACE is not solely related to a flow-limiting stenosis, but more likely relates to the plaque burden and high-risk plaque features along the entire coronary artery tree. Indeed, increasing evidence supports the hypothesis that coronary artery lesions which later cause acute myocardial infarctions often do not narrow the lumen critically [6]. This highlights the need for alternative strategies for patients with SAP to reduce cardiovascular risk and improve quality of life, preferably focusing on improving the cardiovascular system as a whole.

Atherosclerosis is often accelerated by poor medication adherence [7] and a persisting unhealthy lifestyle [8], resulting in recurrent symptoms and cardiovascular events. Therefore, changing lifestyle behaviour should be a central target in the treatment of SAP. As such, cardiac rehabilitation (CR) was shown to improve prognosis, quality of life and medication adherence and reduce hospitalisation rates in patients with coronary heart disease [9]. A meta-analysis showed that contemporary CR in patients with SAP improved exercise capacity and resulted in a reduction of angina [10]. Nonetheless, a 2018 Cochrane review concluded that there is currently insufficient data to validly assess the impact of exercise-based CR on morbidity, mortality and quality of life in this patient group [11]. However, inclusion criteria for patients with SAP were strict, which hampers wider translation, and new studies have since been published.

The aim of this review is first to summarise the physiological mechanisms underlying the potential clinical benefits of exercise-based CR. Secondly, characteristics of exercise-based CR are described, followed by clinical evidence pertaining to the effects of exercise-based CR for SAP. Finally, recommendations for future research are provided. As exercise training is mostly embedded in a comprehensive CR programme, and is not a stand-alone intervention, the term exercise-based CR is used in this review to emphasise the focus on the effect of exercise training. Exercise terms and definitions are described in Tab. 1.

**Table 1:** Exercise terms and definitions

Term	Definition
Exercise	Regular structured physical activity [35]
FITT-principle	Components of exercise prescription: frequency, intensity, time and type [36]
Frequency	Number of exercise sessions per week
*Intensity*	The amount of energy expended per minute of activity, assessed by oxygen uptake per unit of time (ml/min or l/min) or by metabolic equivalent of task [36]
– Light	Activities that require the least amount of effort, with around 57–63% of max HR
– Moderate	Activities that get the heart rate up to 64–76% of max HR
– Vigorous/high	Activities that require the most amount of effort, with 77–95% of max HR
Time	Duration of exercise sessions
*Type*	Modality of exercise activity
– Aerobic	Exercises to improve cardiovascular fitness, such as walking, running or cycling [37]
– Resistance	Exercises to improve muscle strength, e.g. patients training with their own body weight, elastic bands and sports apparatus [37]
– Combined	Both aerobic and resistance training combined
Volume	The total energy expenditure of an exercise programme, determined by the product of session frequency and duration, training intensity, and programme length [36]
Supervised training	Exercise training supervision carried out by adequately trained health professionals, e.g. a physiotherapist [36]
Interval training	Often performed as high-intensity interval exercise. Intermittent, short high-intensity work periods, interspersed with active or passive recovery periods [35, 38]
Continuous training	Higher durations of training of non-variable aerobic activity under moderate-intensity [35, 38]
Multidisciplinary CR	Cardiac rehabilitation programme consisting of several modules, not just including exercise training and physical activity counselling, but also education, risk factor modification, diet/nutritional counselling, and vocational and psychosocial support [36]

*CR* cardiac rehabilitation, *HR* heart rate, *max* maximum

## Physiological adaptations to exercise training

**Fig. 1:**
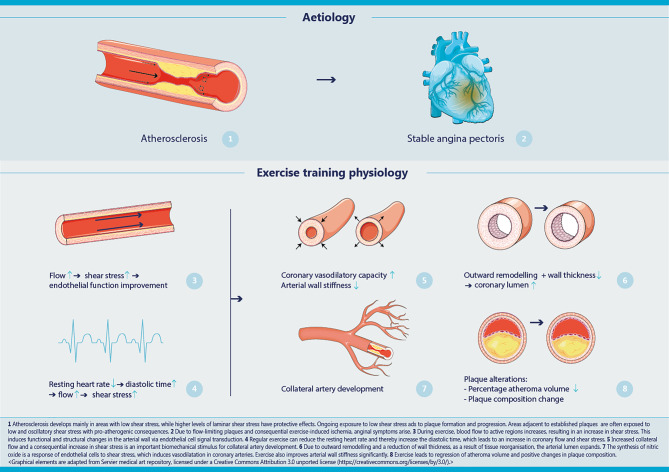
Vascular physiology of exercise training in patients with coronary atherosclerosis

Positive effects of exercise training can be explained by multiple physiological factors [12]. One of these factors is increase in muscle mass and lung capacity, which contributes to increased cardiorespiratory fitness and is associated with a lower cardiovascular risk. In fact, cardiorespiratory fitness is a strong predictor of both cardiac and all-cause mortality in patients with cardiovascular disease (CVD) [13]. For this review, we specifically focus on the effects of exercise-based CR on the vasculature (Fig. 1), including changes in vascular function, vascular structure, atheroma volume and the development of coronary collateral vessels. These effects may be key to reducing symptoms and improving prognosis in patients with SAP.

### Vascular function

The inner surface of a vessel wall is lined with a single semipermeable cell layer, the endothelium. Vascular endothelial dysfunction has an important role in the development of atherosclerosis, as endothelial dysfunction is associated with increased permeability of the vessel wall, inflammation and cell trans-differentiation increase (in which endothelial cells are converted into other cell types), causing atherosclerotic plaque build-up and increased plaque vulnerability [14]. In addition, endothelial dysfunction negatively affects vasodilation capacity and arterial wall stiffness. This highlights the importance of improving endothelial function, for example through medication (e.g., statins) or exercise training [15]. Even when corrected for changes in traditional risk factors, exercise remains an independent predictor of endothelial function [16]. Furthermore, studies have demonstrated that endothelial function, measured using flow-mediated dilation, improves following exercise-based CR in patients with CVD [17].

The beneficial effects of exercise training may relate to an increase in endothelial shear stress (ESS), which is an important contributor to vascular homeostasis and regulates endothelial cell gene expression, morphology and intracellular signalling through specialised mechanosensitive pathways [18]. ESS depends, among other things, on the geometry of the vessel, with low and turbulent shear stress near bifurcations and at the inner curvature of an arch. This is relevant, as atherosclerosis develops mainly in areas with low ESS, while higher levels of laminar shear stress have protective effects [19]. Subsequently, the presence of atherosclerotic plaques may further negatively influence the level and turbulence of shear stress, thereby contributing to additional plaque growth ([20]; Fig. 1). On the other hand, exercise increases blood flow to active regions to meet the increased metabolic demand, leading to elevated ESS. In addition, ESS also increases in non-active regions by thermoregulatory modification of systemic blood flow distribution. These elevations in haemodynamic stimuli are causally linked to functional and structural changes in the arterial wall via endothelial cell signal transduction [12]. For example, the synthesis of nitric oxide induces vasodilatation and antithrombotic endothelial mechanisms. Repetitive increases in ESS induced by a 4-week moderate-intensity exercise training programme in patients with CVD were shown to improve endothelium-dependent vasodilatation in coronary arteries and to enhance nitric oxide bioavailability [12]. This is mediated by an increased expression and activation of endothelial nitric oxide synthase.

In conclusion, exercise training is associated with improved endothelial function, which seems to be related to better clinical outcomes and is mechanistically driven through repeated exposure to ESS. More importantly, these improvements in vascular function may translate to health benefits in SAP.

### Vascular structure

Functional adaptations, as discussed in the previous paragraph, precede most structural adaptations [12]. A property of arteries, influenced by both functional and structural vascular health, is arterial wall stiffness, which can be measured via pulse wave velocity and is a marker for cardiovascular events [21]. Aerobic exercise significantly lowers measures of arterial stiffness and this effect seems amplified with higher exercise intensity [22].

An important structural adaptation induced by exercise training is the increase in coronary artery size [23, 24]. Repetitive exposure of coronary arteries to elevations in blood flow, and therefore ESS, leads to significant outward remodelling, with nitric oxide as an important mediator. Another structural adaptation typically observed following prolonged periods of exercise training is a systemic decrease in wall thickness [25], which is reflective of the atherosclerotic process. Due to outward remodelling and a reduction in wall thickness, as a result of tissue reorganisation, the arterial lumen expands. This facilitates an increase in blood supply during exercise when cardiac oxygen demand is high, also to coronary segments located distal to obstructive plaques. Such effects may contribute to reducing symptoms.

### Arteriogenesis

Another possible mechanism leading to symptom reduction is collateral coronary artery development, which becomes increasingly important in supplying blood to the myocardium in the presence of arterial obstruction. The obstruction leads to an increased pressure gradient, contributing to increased flow, and thus ESS, through collateral arteries. These haemodynamic stimuli promote artery development (arteriogenesis), which is a self-limiting process until the pressure gradient is normalised by the expanding lumen of the collateral artery. To drive further collateral artery development, the pressure gradient can be reinforced by increasing the coronary flow as a response to higher myocardial oxygen demand during exercise [26].

Another mechanism contributing to arteriogenesis is a reduction of the resting heart rate, which increases the length of diastole. As most coronary blood flow occurs in diastole, this leads to increased shear stress and subsequent arteriogenesis. To support this concept, an RCT in 46 patients with chronic coronary syndrome showed that ivabradine (which reduces the heart rate by inhibiting the electrical current in the sinoatrial node) led to a significant increase in the collateral flow index compared with placebo after 6‑months of follow-up [27]. In addition, another RCT demonstrated a significant improvement in the collateral flow index in response to moderate- and high-intensity exercise after 4 weeks [28]. Taken together, these data suggest exercise induces coronary arteriogenesis, which may contribute to limiting or attenuating symptoms and to myocardial protection.

### Atheroma characteristics

The risk of acute and recurrent coronary events in patients with CVD is mainly related to their coronary atheromatous plaque volume and composition [29, 30]. Indeed, each 1% reduction in percent atheroma volume (PAV) is associated with a 20% reduction in the odds of MACE [31]. Importantly, the PREDICTION study showed that low ESS precipitates progression in PAV [30], suggesting that exercise training may reduce PAV. Indeed, Madssen et al. found a modest reduction in coronary necrotic core following exercise training, independent of exercise intensity [32]. Similarly, other studies using invasive techniques to characterise individual plaque features, such as intravascular sonography and optical coherence tomography, also demonstrated a correlation between exercise volume and a reduction in plaque and lipid volume [33], and that PAV and total atheroma volume significantly decreased after 6 months of supervised high-intensity interval training [34]. In this latter study, LDL-cholesterol and Apo‑B levels were unchanged during the study period, making it unlikely that changes in cholesterol explain the change in atheroma volume. The regression of coronary atheroma volume may be explained by increased coronary ESS. In conclusion, although there is only recent interest in this field, increasing evidence suggests positive effects of exercise on plaque characteristics, which may relate to both symptom and residual risk reduction.

## Exercise-based CR: role of exercise characteristics

Whereas exercise-based CR comes in different forms and intensities, the characteristics of the optimal exercise training programme for SAP are still inconclusive. Multiple factors, described by the FITT-principle (frequency, intensity, time and type) may determine the effects on cardiorespiratory fitness (CRF), and therefore possibly health-related outcomes.

Pertaining to exercise intensity, a 2021 meta-analysis comparing the effect of different exercise intensities on CRF (VO2peak) showed moderate-to-vigorous and vigorous exercise were the most effective intensities to enhance CRF [38]. Similarly, meta-analyses show high-intensity interval training to be superior to moderate-intensity continuous training in improving CRF and cardiovascular risk factors [35, 39]. However, further research is needed to assess the safety and effect of higher training intensities, particularly because patients with SAP are underrepresented in previous studies. Although evidence does not indicate that supervised training on or above the ischaemic threshold is unsafe [40], the European Association of Preventive Cardiology advises training intensity between the first and the second ventilatory threshold.

Frequency and duration of exercise bouts are typically combined into the amount or volume of exercise, which is often presented against the risk of cardiovascular events through a curvilinear dose-response relation. The largest relative health benefit is achieved in patients who change from being inactive to some activity, while each additional increase in physical activity leads to a smaller health benefit, eventually leading to a plateau. When focusing on possible adverse effects of physical activity, a large observational cohort study found that large volumes of exercise were associated with more coronary plaques [41]. Although work suggested this may be driven by the vigorous intensity, rather than volume per se [42], training intensities and volumes were not objectively measured and the effect of these adaptations on the risk of MACE is still undefined.

The two most commonly applied exercise training modalities relate to aerobic and resistance training. Aerobic training is typically classified in light-, moderate- and high-intensity training, as discussed in more detail above, and is strongly linked to improvements in cardiorespiratory fitness. In addition, resistance training consists of repetitive exercise bouts and is aimed to improve muscle strength and volume. Given their distinct working mechanisms and effects, it is not surprising that studies support the combination of aerobic and resistance training rather than aerobic training alone to achieve the greatest benefits [37]. Contemporary exercise-based CR indeed includes both types of exercise.

Taken together, the FITT-principles are important factors in designing exercise programmes. Whilst the optimal combination of FITT has not been specified, an emerging challenge of CR and follow-up cardiac care is the continued adherence to a physically active and healthy lifestyle. The optimal approach to achieve this is still unresolved, but may include a more personalised approach, extended guidance after CR, or the possibility of repeat prescriptions. Based on the ‘exercise is medicine’ principle, an important consideration is that, as opposed to most medicine, exercise is largely free of adverse effects.

## Clinical benefits of exercise-based CR

As shown above, exercise has beneficial, dose-dependent health effects, including in CVD [43, 44]. Higher physical activity levels are associated with a lower risk of death and cardiovascular events, irrespective of country and income [43]. Besides the benefits of exercise training as adjuvant therapy for various disease states, exercise training has also proven to be clinically and economically beneficial as initial therapy, for example for intermittent claudication [45, 46]. Given that both intermittent claudication and SAP share the systemic involvement of atherosclerosis in their pathophysiology, exercise training could also be a beneficial therapy for patients with SAP.

In coronary artery disease, and SAP specifically, studies investigated the effect of exercise-based CR as adjuvant therapy after revascularisation. An observational cohort study using Dutch health insurance data showed a 31% lower risk of all-cause mortality in CR participants compared with nonparticipants with SAP, both with or without revascularisation [47]. This health benefit in SAP is in agreement with the overall 32% lower risk of all-cause mortality associated with CR in this study. Another Dutch population-based cohort study showed that multidisciplinary CR is associated with a substantial 4‑year survival benefit, an effect that was present regardless of the type of intervention or diagnosis (i.e. acute coronary syndrome, SAP) [48]. Moreover, a prospective registry analysis in patients with long coronary artery lesions (~70% SAP) showed that CR resulted in 35% less late luminal loss in the stented segment after 9 months compared with the control group [49]. Taken together, these data suggest that exercise-based CR as an adjuvant therapy following PCI is associated with better survival and a lower risk of MACE.

However, only a few studies have explored exercise-based CR as initial therapy for SAP and directly compared results against revascularisation. A recent retrospective cohort study evaluated health insurer data of 18, 383 patients with chronic coronary syndrome and found that prescription of CR (without revascularisation) is associated with a significantly lower risk of all-cause mortality and acute myocardial infarction compared with revascularisation alone [50]. Whilst these retrospective analyses provide insight, RCTs are needed to directly evaluate effects of exercise-based CR in SAP. To our knowledge, Hambrecht et al. published the only RCT comparing PCI with daily exercise training over a period of 1 year and found a significantly lower event rate and higher exercise capacity in favour of the exercise group [51]. These benefits persisted after 2‑years of follow-up. Taken together, studies suggest superior clinical outcomes following exercise-based CR compared with revascularisation as a primary treatment for SAP, although contemporary, properly designed and powered RCTs are warranted to confirm these observations.

## Future directions

### Evaluate clinical impact

The impact of exercise-based CR on mortality and morbidity in SAP is still not well established. Although prospective observational studies suggest beneficial effects of exercise-based CR, either as an initial strategy or adjuvant to revascularisation, the field is lacking sufficiently powered RCTs. Supported by a previous, underpowered RCT [51] and a retrospective study [50], the necessity for well-executed randomised trials comparing exercise-based CR with revascularisation is evident. Such trials need to report MACE, risk-factor management, symptoms of angina and health-related quality of life. These should also assess cost-effectiveness and recruit participants who are reflective of the general angina population.

### Optimising the CR programme

The key question relates to the optimum exercise-based CR protocol that will lead to the most favourable clinical results. In addition, when used as a primary treatment for SAP, the safety of exercise is an important consideration, especially since flow-limiting stenosis could still be present. Particularly since training intensity and volume contribute to the positive effect of exercise training, studies are needed to assess both effectivity and safety of high exercise intensities. Furthermore, to preserve long-term results, future research should focus on strategies to maintain physical activity levels and to achieve sustainable changes in lifestyle. Finally, it is important to consider that patients may differ in physiological responses and adaptation to exercise training, but may also have personal preferences, highlighting the need for personalised exercise prescriptions to optimise the results.

### Understanding underlying mechanisms of exercise-based CR

For future studies it is important to understand the effects of exercise-based CR on endothelial function, structural adaptation of arteries, coronary arteriogenesis and plaque volume and composition, since this may contribute to answering the aforementioned key questions relevant in SAP. Understanding how exercise-based CR characteristics, but also subject characteristics, interfere with the physiological adaptations may contribute to optimising the exercise protocol. Such optimisation may ultimately translate to optimised clinical benefits. Furthermore, the interaction between the physiological pathways and the impact of different forms and intensities of exercise remain largely unknown. New studies focussing on coronary artery physiology and the effect of different training programmes, preferably in combination with clinical endpoints, will give a better understanding of the optimal training conditions for patients with SAP.

## Conclusion

Preliminary studies have provided provocative data supporting the idea that exercise-based CR has the potential to complement and possibly even replace revascularisation as a first-line treatment for SAP in addition to optimal medical therapy. For this reason, high-quality, randomised trials are needed to assess the physiological and clinical benefits of exercise-based CR for patients with SAP. In achieving maximal benefits, both in the short and the long term, investigators should consider comparing different forms of exercise training and should focus on prolonged guidance to optimise long-term adherence to improved physical activity levels. Pertaining to implementation and dissemination of this knowledge, it should be noted that changing clinicians’ views and long-standing paradigms is a great challenge since revascularisation therapy is widely assumed to be a quick and effective strategy to achieve symptom reduction, despite the high costs and the current debate on its effect on prognosis and clinical benefits. Taken together, it is time to view exercise as medicine for the management of SAP and in this attempt it is important to closely involve clinicians, patients and other stakeholders to optimise and implement this novel care pathway.
